# Dedifferentiated liposarcoma with leukocytosis. A case report of G-CSF-producing soft-tissue tumors, possible association with undifferentiated liposarcoma lineage

**DOI:** 10.1186/1477-7819-5-131

**Published:** 2007-11-16

**Authors:** Akio Sakamoto, Hiroshi Matono, Tatsuya Yoshida, Kazuhiro Tanaka, Shuichi Matsuda, Yoshinao Oda, Yukihide Iwamoto

**Affiliations:** 1 Department of Orthopedic Surgery, Graduate School of Medical Sciences, Kyushu University, Fukuoka, 812-8582, Japan; 2 Department of Anatomic Pathology, Graduate School of Medical Sciences, Kyushu University, Fukuoka, 812-8582, Japan

## Abstract

**Background:**

Granulocyte-colony-stimulating factor (G-CSF) functions as a hematopoietic growth factor and it is responsible for leukocytosis. G-CSF-producing tumors associated with leukocytosis include various types of malignancies.

**Case presentation:**

We report the case of a 72-year-old man with dedifferentiated liposarcoma characterized by dedifferentiated components of malignant fibrous histiocytoma (MFH)-like features in addition to well-differentiated lipoma-like liposarcoma, arising from his upper arm. Preoperative laboratory data showed leukocytosis (103,700/μl). The serum level of G-CSF was also elevated (620 pg/ml [normal, <8 pg/ml]). Nine days after the surgery, the leukocytosis was relieved (WBC; 6,920/μl) and the elevated serum G-CSF level was significantly decreased (G-CSF; 12 pg/ml). One month after the surgery, leukocytosis gradually began to appear again. Three months after the surgery metastatic lung lesions were confirmed, and the patient subsequently died of respiratory problems. In the English literature regarding soft-tissue tumors with leukocytosis, including the current case, we could review a total of 6 cases of liposarcoma with leukocytosis. The subtype of these 6 liposarcoma cases was undifferentiated liposarcoma, comprising dedifferentiated liposarcoma in 4 cases and pleomorphic liposarcoma in 2 cases.

**Conclusion:**

Since the only other soft-tissue tumor that was associated with leukocytosis was MFH, and since MFH is characterized by the absence of any specific differentiation, we would like to propose a possible association between G-CSF-producing soft-tissue tumors and an undifferentiated liposarcoma lineage, such as dedifferentiated liposarcoma or pleomorphic liposarcoma.

## Background

Granulocyte-colony-stimulating factor (G-CSF) enhances differentiation along the neutrophil lineage, and accelerates maturation of metamyelocytes into mature neutrophils. Consequently, G-CSF is known to function as a hematopoietic growth factor and it is known to be responsible for leukocytosis. G-CSF-producing tumors associated with leukocytosis include various types of malignancies. In epithelial cancers, the expression of G-CSF has been associated with poor differentiation and invasiveness [1-3]. However, it is a rare event among soft-tissue tumors for leukocytosis to be associated with an elevated level of serum G-CSF. Furthermore, although malignant fibrous histiocytoma (MFH), which is characterized by the absence of any distinct differentiation, has been reported to be accompanied by leukocytosis [4-6], this is extremely rare in the case of soft-tissue tumors with specific differentiation.

## Case presentation

**Figure 1:**
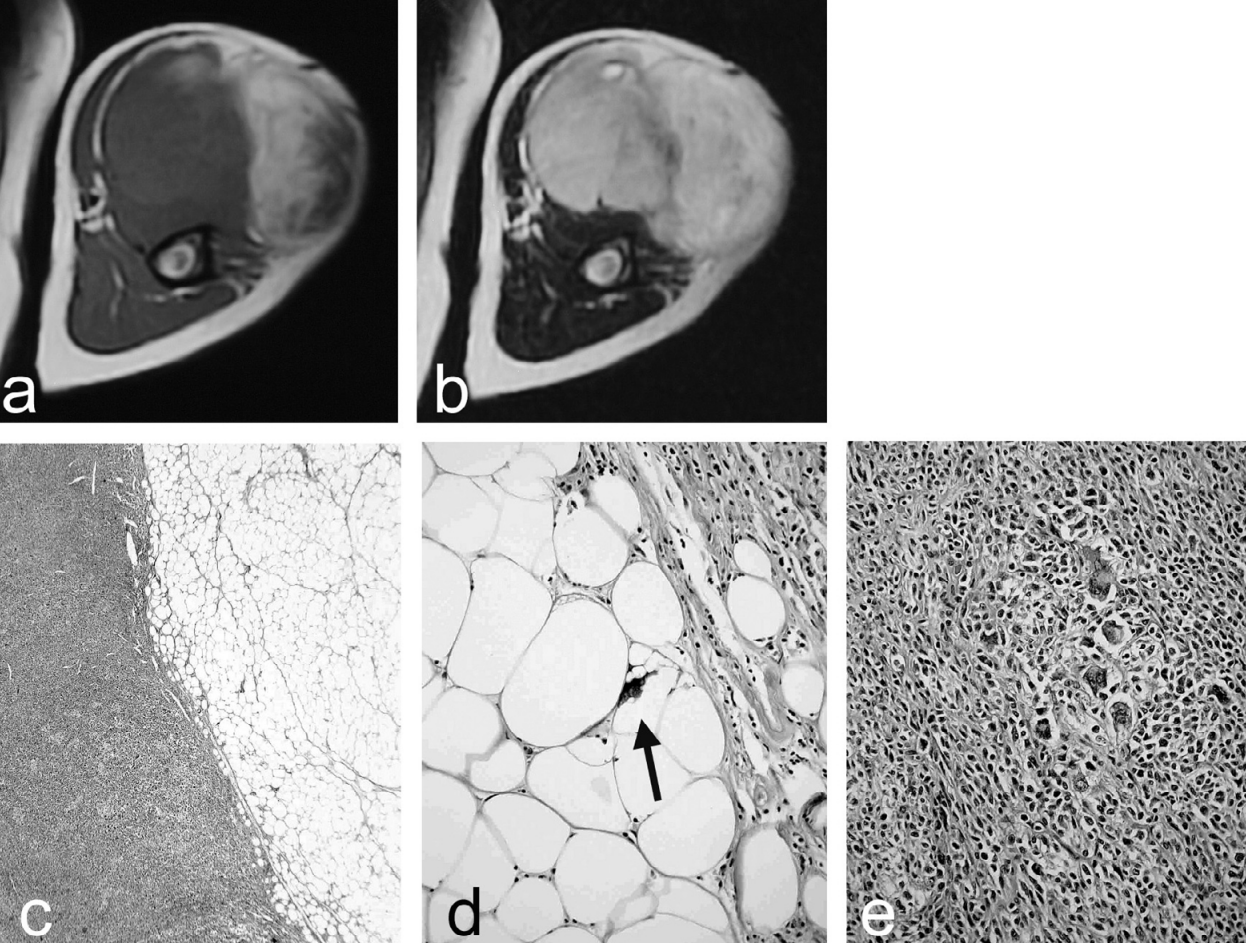
MRI shows a tumor with two components of high T1-weighted (a) and high T2-weighted images (b) (right portion; well-differentiated components), and of low T1-weighted (a) and high T2-weighted images (b) (left portion; dedifferentiated components). Well-differentiated components and dedifferentiated components with a distinct border (c). Well-differentiated components show well-differentiated lipoma-like liposarcoma with lipoblasts (arrow) (d). Dedifferentiated components are composed of atypical spindle cells in short fascicles, resembling MFH (bottom; left) (e).

A 72-year-old man who suffered from a tumor in his upper arm presented to us (Figure 1a, 1b). Body temperature was 38.1°C. Histological diagnosis of the resected tumor was dedifferentiated liposarcoma characterized by a well-differentiated lipoma-like liposarcoma component (Figure 1c, 1d) and a dedifferentiated component with MFH-like features (Figure 1e).

**Figure 2:**
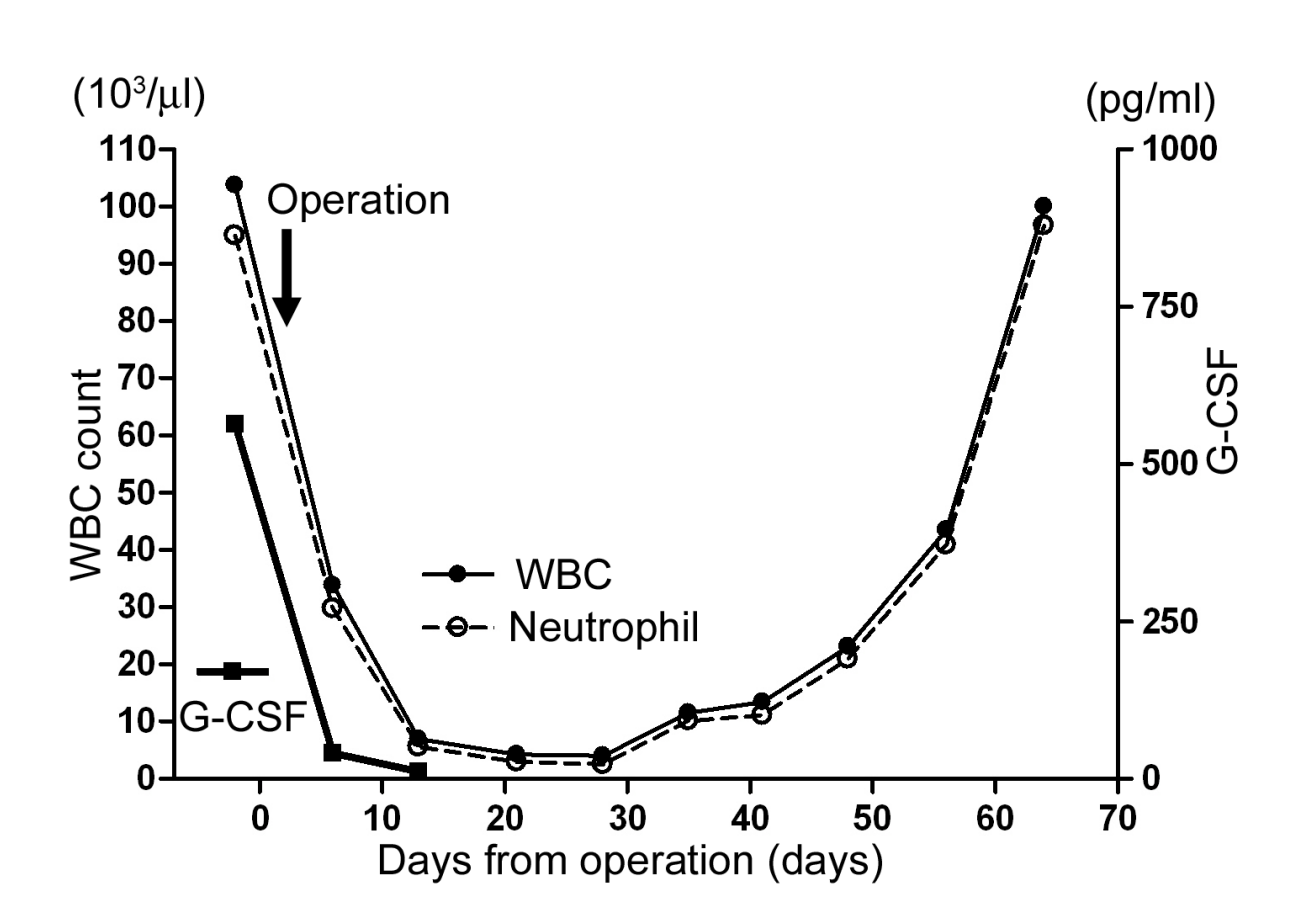
Time course of white blood cell count and serum G-CSF level. The count of white blood cells including neutophils, and the serum level of G-CSF decreased after surgery.

The blood data of white blood cells and serum level of G-CSF are summarized in Figure 2 and Table 1. Preoperative laboratory data showed leukocytosis (103,700/μl) predominantly in the neutrophils (neutrophils; 91.5% [normal, 40–70%]). The serum level of G-CSF was also elevated (620 pg/ml [normal, <8 pg/ml]). Leukocytosis was relieved (white blood cells; 33,800/μl [neutrophils; 88.0%] and 6,920/μl [neutrophils; 80.6%]) and the elevated serum G-CSF level was significantly decreased (G-CSF; 44 pg/ml and 12 pg/ml), 2 and 9 days after the surgery, respectively. One month after the surgery leukocytosis gradually began to appear again. Three months after the surgery, metastatic lung lesions were confirmed, and the patient subsequently died of respiratory problems.

**Table 1:** Summary of blood data

Days from OPE	-4	2	9	17	24	31	37	44	52	60
WBC (×10^3^)	103.7	33.8	6.92	4.2	4.0	11.5	13.4	23.1	43.5	100.0
Neutrophils (%)	91.5	88.0	80.6	69.1	61.5	87.5	83.2	90.6	93.7	96.6
G-CSF(pg/ml) [normal, <8 pg/ml]	620	44	12	NA	NA	NA	NA	NA	NA	NA

OPE; operation, WBC; white blood cells, G-CSF; granulocyte-colony-stimulating factor, NA; not assessed

Expression of G-CSF (anti-G-CSF [Ab1], Calbiochem, San Diego CA, USA) failed to be detected not only in the well-differentiated lipoma-like liposarcoma components, but also in the dedifferentiated components, immunohistochemically (data not shown).

## Discussion

The leukocytosis seen in the current case with dedifferentiated liposarcoma seemed to be due to stimulation by the tumor-produced G-CSF, on the basis that both the leukocytosis and the elevated level of G-CSF disappeared after the tumor was resected. In a previous report, leukocytosis appeared at an advanced stage, as characterized by poorly differentiated adenocarcinoma, not in the early stage, as characterized by well-differentiated adenocaricnoma, in the same lesion of gastric cancer. In that gastric cancer case, immunopositivity of G-CSF was only seen in the poorly differentiated adenocarcinoma, not in the well-differentiated adenocarcinoma. It is thus possible that histological changes may influence the acquisition of G-CSF-producing ability [7]. In the current study, immunohistochemical study failed to detect any expression of G-CSF. It would seem that G-CSF-producing ability was acquired during the course of tumor progression, because leukocytosis appeared after noticeable tumor growth. It is also possible that the components of G-CSF-producing cells are heterogeneous in the tumor. Neoplastic cells in unexamined histological sections may express G-CSF, and an immunohistochemical study would have detected G-CSF expression.

In the English literature, including the current case, there have been 6 cases of liposarcoma accompanied by leukocytosis and associated with G-CSF expression [8-12]. Gender distribution was 3 males and 3 females, while the average age was 64.7 (range; 50–77) years. The sites were the following; retroperitoneum in 4 cases, mesenterium in 1 case and upper arm in 1 case. The subtype of these liposarcoma cases was dedifferentiated liposarcoma in 4 cases [10-12] and pleomorphic liposarcoma in 2 cases [8,9]. Pleomorphic liposarcoma showed the features of pleomorphic sarcoma, mimicking MFH, and at least focally contained typical multivacuolated lipoblasts. In the case of epithelial tumors, G-CSF-producing tumors have been reported to be associated with poor differentiation [1-3]. Up until now, there have been no reported cases of well-differentiated liposarcoma with leukocytosis. Therefore, it may also be true that G-CSF expression is associated with undifferentiated liposarcoma, including both dedifferentiated liposarcoma and pleomorphic liposarcoma. Furthermore, it might be better to search for dedifferentiated components in an effort to provide a possible diagnosis of dedifferentiated liposarcoma, when well-differentiated liposarcoma is accompanied by leukocytosis.

MFH has been reported to be accompanied by leukocytosis, previously [4-6]. The diagnostic criteria of MFH are defined as "pleomorphic spindle cell sarcoma with no distinct line of differentiation". The diagnosis of MFH is commonly made by exclusion of other definitive sarcomas, such as liposarcoma and leiomyosarcoma. Recently, after reassessment of pleomorphic sarcomas, the tumors in a certain number of cases have been proved to be specific sarcomas other than MFH. Therefore, there is the possibility that the reported case of MFH with leukocytosis actually had a liposarcoma lineage. In addition, among soft-tissue tumors with specific differentiation, there seem to be no reported cases with leukocytosis, besides liposarcoma. The association between G-CSF production and undifferentiated liposarcoma formation implies that they share the same signaling pathways in part.

In epithelial cancers, G-CSF expression has been associated with poor differentiation and invasiveness [1-3]. Moreover, G-CSF expression was reported to be linked to poor prognosis in patients with lung carcinoma [13]. G-CSF has been shown to stimulate tumor cell growth and migration in vitro [14-17]. In a similar manner, G-CSF may have contributed to the aggressive behavior of dedifferentiated liposarcoma in the current case. A transcription factor STAT3 is a key mediator of G-CSF signaling. STAT3 potently promotes proliferation, survival, invasion and angiogenic effects through regulation of multiple gene products [18-21]. Survivin is one of the STAT3-regulated genes, and has an important function in regulating the growth of cancer cells [22]. It has been indicated that G-CSF potently activates STAT3 and STAT3-dependent survivin expression in bladder cancer cells [23]. Further examination would be necessary to confirm whether or not this mechanism holds true for liposarcoma.

## Conclusion

In this report, we present a case of dedifferentiated liposarcoma with leukocytosis. The leukocytosis seemed to be associated with an elevated level of serum G-CSF. We would like to propose a possible association between G-CSF-producing soft-tissue tumors and an undifferentiated liposarcoma lineage, such as dedifferentiated liposarcoma or pleomorphic liposarcoma.

## Competing interests

The author(s) declare that they have no competing interests.

## Authors' contributions

**AS **drafted the manuscript. **HM **performed the immunohistochemical study. **TY**, **HM**, **KT**, **SM **and **YO **participated in the design of the study. **YI **conceived of the study, and participated in its design and coordination and helped to draft the manuscript. All authors read and approved the final manuscript.
